# Supplementary material for: Exploring Volatile Organic Compounds in Rhizomes and Leaves of Kaempferia parviflora Wall. Ex Baker Using HS-SPME and GC–TOF/MS Combined with Multivariate Analysis
Source: Metabolites. 2023 May 11;13(5):651. doi: 10.3390/metabo13050651 (PMC10223059; doi:10.3390/metabo13050651)
Supplement: Supplementary file 1 [file metabolites-13-00651-s001.zip › metabolites-2347588-supplementary.pdf]

Supporting Information:

The PCA was performed to provide a graphical overview and to visualize the differences between examined *K. parviflora* samples. The result showed clear separation of four groups, accounting a total variance of 69.6% (PC1, 49.1%; PC2, 20.5%). The loading plots were generated from PCA to determine the variables responsible for separating the data, and the result was shown in supplementary Figure S1. The loadings plot shows corresponding variation of VOCs among the different groups. The representative chromatograms showing the identified VOCs from rhizome samples, leaf samples and EPA standard are shown in Figure S2.

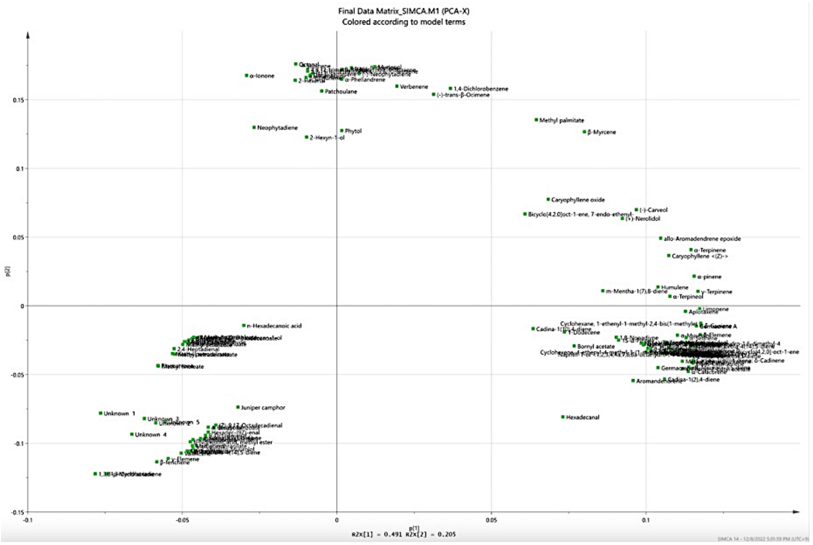

Figure S1. Loading score plot of Principal Component Analysis (PCA) on rhizomes and leaves of *K. parviflora* from field and growth room conditions (n = 24). The first two principal components (PC1, PC2) accounted for a total of 69.6% of the variance.

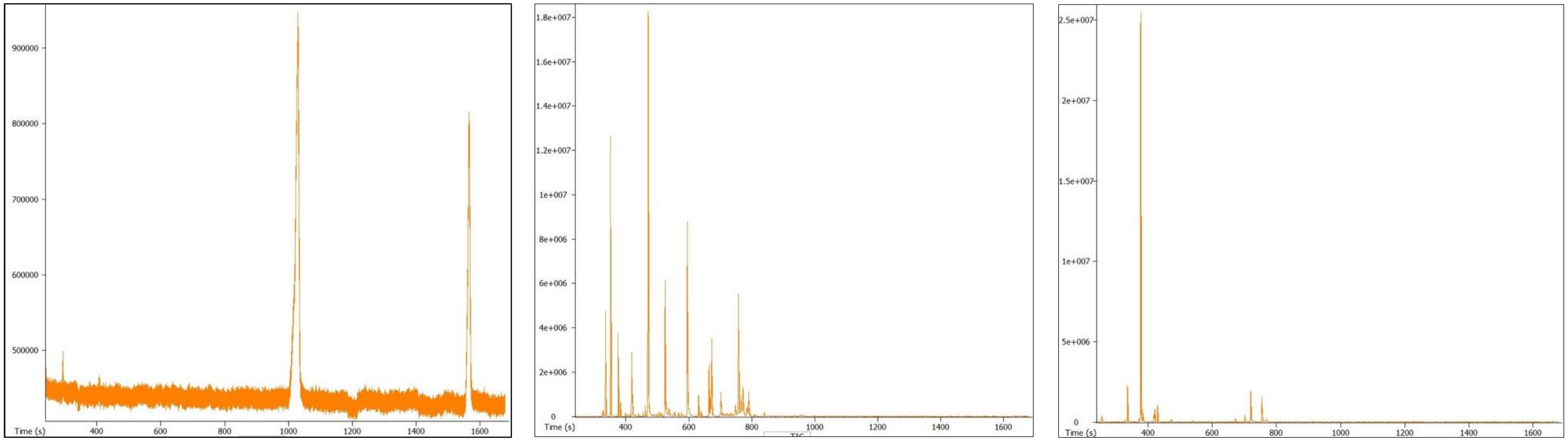

(A) (B) (C)

Figure S2. The representative chromatograms showing the identified VOCs : EPA standard (A), rhizome samples (B), leaf samples (C)
